# Postoperative analgesic effect, of preoperatively administered dexamethasone, after operative fixation of fractured neck of femur: randomised, double blinded controlled study

**DOI:** 10.1186/s12871-016-0247-5

**Published:** 2016-09-22

**Authors:** Szilard Szucs, David Jessop, Gabriella Iohom, George D. Shorten

**Affiliations:** 1 Department of Anaesthesia, Intensive Care and Pain Medicine/University College Cork, Cork University Hospital, Wilton, Cork, Ireland; 2 Faculty of Medicine, Henry Wellcome Laboratories for Integrative Neuroscience and Endocrinology, University of Bristol, Bristol, UK

**Keywords:** Analgesics, Dexamethasone, Intravenous injections

## Abstract

**Background:**

Fractured neck of femur is a common cause of hospital admission in the elderly and usually requires operative fixation. In a variety of clinical settings, preoperative glucocorticoid administration has improved analgesia and decreased opioid consumption. Our objective was to define the postoperative analgesic efficacy of single dose of dexamethasone administered preoperatively in patients undergoing operative fixation of fractured neck of femur.

**Methods:**

Institutional ethical approval was granted and written informed consent was obtained from each patient. Patients awaiting for surgery at Cork University Hospital were recruited between July 2009 and August 2012. Participating patients, scheduled for surgery were randomly allocated to one of two groups (Dexamethasone or Placebo). Patients in the dexamethasone group received a single dose of intravenous dexamethasone 0.1 mg kg ^-1^ immediately preoperatively. Patients in the placebo group received the same volume of normal saline. Patients underwent operative fixation of fractured neck of femur using standardised spinal anaesthesia and surgical techniques. The primary outcome was pain scores at rest 6 h after the surgery.

**Results:**

Thirty seven patients were recruited and data from thirty patients were analysed. The groups were similar in terms of patient characteristics. Pain scores at rest 6 h after the surgery (the principal outcome) were lesser in the dexamethasone group compared with the placebo group [0.8(1.3) vs. 3.9(2.9), mean(SD) *p* = 0.0004]. Cumulative morphine consumption 24 h after the surgery was also lesser in the dexamethasone group [7.7(8.3) vs. 15.1(9.4), mean(SD) mg, *p* = 0.04].

**Conclusions:**

A single dose of intravenous dexamethasone 0.1 mg kg ^-1^ administered before operative fixation of fractured neck of femur improve significantly the early postoperative analgesia.

**Trial registration:**

ClinicalTrials.gov identifier: NCT01550146, date of registration: 07/03/2012

## Background

Fractured neck of femur (FNF) is a common cause of hospital admission in the elderly and requires operative fixation with an associated mortality of 5–8 % at 3 months [1].

Dexamethasone is a synthetic adrenocortical steroid, used widely in anaesthesia as an antiemetic in surgical patients. Dexamethasone inhibit cortisol secretion by inhibiting the hypothalamo-pituitary-adrenal axis. At the pituitary level inhibition involving changes in transcription [2, 3]. Cortisol exists in free (unbounded) and protein-bound forms in serum but only in a free form in saliva. The free form is the biologically active one [4]. Glucocorticoids are inhibitors of both phospholipase and cyclooxygenase enzyme, intravenously administered dexamethasone 16 mg provides prolonged postoperative analgesia from 24 to 72 h after breast surgery [5]. Preoperative dexamethasone reduce postoperative nausea, has a prolonged suppressive effect on the inflammatory response and decreases dynamic pain 24 h after total hip arthroplasty [6]. Administration of i.v. dexamethasone prior to intrathecal meperidine injection enhance postoperative analgesia and reduces postoperative nausea and vomiting [7].

Recently were published two editorials which were emphasised the potential side effects of single dose of dexamethasone [8, 9], however the authors agreed that further evidence needed to justify to not administer dexamethasone in patients with low risk for the side effects.

Single dose dexamethasone has not been evaluated for its effects on postoperative pain and the inflammatory response to surgery in surgical fixation of FNF.

In this study, our hypothesis was that preoperative administration of a single dose of dexamethasone 0.1 mg kg ^-1^ enhances postoperative analgesia 6 h after the surgery in patients undergoing operative fixation of FNF. Previous clinical studies used dexamethasone from low [7] dose to high dose [6]. Taking into consideration the anticipated age profile of our patients and the associated lesser metabolic rate we selected a lesser dose, 0.1 mg kg ^-1^, of dexamethasone. The primary outcome parameter was pain score at rest 6 h after surgery.

## Methods

With institutional ethical approval [Clinical Research Ethics Committee of the Cork Teaching Hospitals [(ECM 3 (bbbb) 07/07/09.)] and having retrospectively registered with ClinicalTrials.gov (NCT01550146), a prospective, double-blind, randomised, placebo-controlled trial was undertaken at Cork University Hospital, Cork, Ireland between July 2009 and August 2012. Patients were randomly assigned to receive either preoperative single dose of dexamethasone or the same volume of normal saline as placebo (Dexamethasone D or Placebo P). We used a random number tables and sealed envelopes prepared by one of the co-author (GI). Based on the randomisation the solution was prepared by a different anaesthetist and was administer by a blinded anaesthesia provider not involved in the study. The anaesthetist and the independent observer were both blinded. Written, informed consent was obtained from all patients.

Patients admitted on to the Emergency Department at Cork University Hospital with FNF, American Society of Anaesthesiologists grades I -III and aged >65 years, were invited to participate in the study. Exclusion criteria were endocrine disorders (including I and II type of Diabetes mellitus), prior diagnosis of depression, corticosteroid treatment (in any form) within the previous last 4 months, Mini-Mental Score < 22, coagulation disorders, head injury, other associated injuries, loss of consciousness, renal dysfunction and sepsis.

Patients in group D received a single dose of dexamethasone 0.1 mg kg ^-1^ (1 mg ml ^-1^) i.v. on arrival to the operating theatre (prior to performance of a femoral nerve block to facilitate positioning for spinal anaesthesia). Patients in group P received the same volume 0.1 ml kg ^-1^ of normal saline i.v on arrival to the operating theatre.

On arrival to the operating theatre, an 18 G cannula was inserted for i.v. drugs and fluid administration. Ultrasound-guided femoral nerve block (15 mls of 2 % lignocaine) was performed to facilitate positioning for spinal anaesthesia, which was performed using hyperbaric bupivacaine (11 mg in patients < 70 kg and 12.5 mg in patients > 70 kg) through a 25 G spinal needle.

All patients received paracetamol 1 g i.v. during surgery and six hourly thereafter for a week. Rescue analgesia consisted of 5–10 mg morphine i.m.

An independent blinded observer assessed pain severity at rest and on movement using a numeric rating score (NRS) (0–no pain; 10-worst pain imaginable) on arrival to the operating theatre, on arrival to recovery, and at 6, 12, 24, 48, 72 h and 1 week post-operatively. Salivary cortisol level was measured by radioimmunoassay [10].

Patients were evaluated for Nausea/Vomiting, sedation and pruritus (4 on a observational scale 1–4) by the independent blinded observer at the same time points.

Analgesic consumption were also recorded.

### Statistical analysis

Quantitative data were tested for normality and, as appropriate, analysed using the unpaired two-tail Student *t* test. Categorical data were examined using Chi-squared test (or Fisher’s Exact Test as appropriate). *P* < 0.05 was considered significant.

Power analysis was performed based on the data published in a previous publication [11] which correlates with our previously published data [12]. To detect a 50 % decrease in pain scores 6 h after the surgery, with a two-sided 5 % significance level and a power of 80 %, it was calculated that the minimum sample size was 17 patients per group.

## Results

**Fig. 1:**
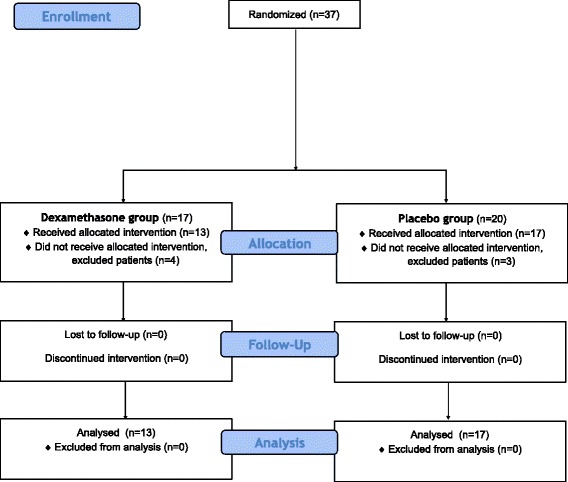
Flow diagram, showing data about enrollment, allocation, follow-up and statistical analysis

Thirty seven patients were recruited to this study of whom 30 were managed per protocol. Seven patients were excluded: in two cases, the patients’ mental status deteriorated after recruitment and in five cases, it was elected to perform the surgery under general anaesthesia for reasons unrelated to the study (Fig. 1). In the dexamethasone group one patient was excluded from the analysis because of mental status deterioration after recruitment and in three cases, surgery was performed under general anaesthesia for clinical reasons independent of the study. Patient characteristics were similar in the two groups (Table 1).

**Table 1:** Patient Characteristics

	Dexamethasone group	Placebo group	*p* value
Gender (Female/Male)	8/5	12/5	
Age (years) mean (SD)	74 (20.3)	71.9 (19.5)	0.38
BMI (kg/m2) mean (SD)	21.4 (7.2)	23.4 (7.4)	0.18
Fractured side (Right/Left)	6/7	7/10	

Data displayed as number of cases. *BMI* Body Mass Index

**Fig. 2:**
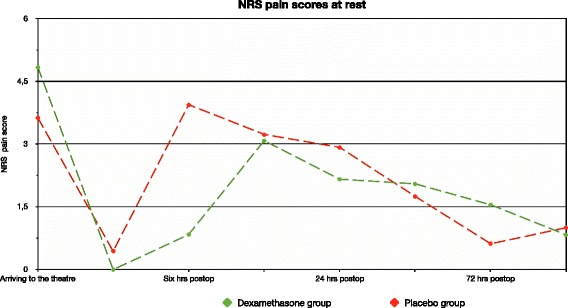
Showing Numeric rating scale (NRS) pain scores at rest in the first postoperative week. Primary outcome is NRS pain score 6 h after the surgery

**Fig. 3:**
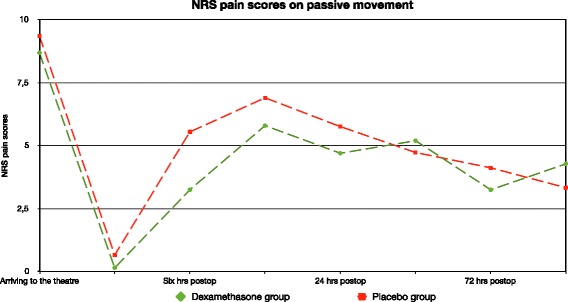
Showing Numeric rating scale (NRS) pain scores on passive movement in the first postoperative week

**Fig. 4:**
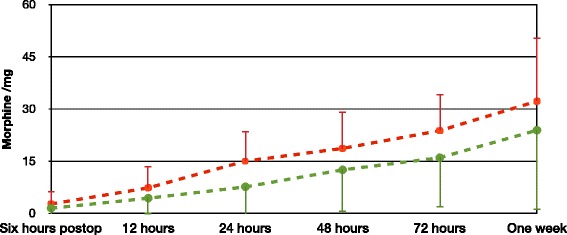
Showing cumulative morphine consumption in the first postoperative week. Placebo group (*red*), Dexamethasone group (*green*)

The pain scores at rest 6 h after the surgery were lesser in the dexamethasone group compared with the placebo group [0.8(1.3) vs. 3.9(2.9), mean(SD) *p* = 0.0004] (Fig. 2). Pain scores on passive movement six hours after the surgery tended to be lesser in the dexamethasone group [3.2(2.6) vs. 5.5(3.8), mean(SD) *p* = 0.055] (Fig. 3) although this did not achieve statistical significance. Cumulative morphine consumption 24 h after the surgery (mg) was lesser in the dexamethasone group than in the placebo group [7.7(8.3) vs. 15.1(9.4), mean(SD) respectively; *p* = 0.04] (Fig. 4).

Cortisol concentration (ng/ml) measured in saliva was significantly lesser in the dexamethasone group on the first postoperative evening [0.7(0.8) vs. 3.8(3.4), mean(SD); *p* = 0.01].

Pain scores measured at rest and on passive movements at the other time points studied were similar in the two groups (Figs. 2 and 3).

Compared with those in the placebo group, patients in the dexamethasone group had lesser systolic blood pressure (mmHg), 6 h postoperatively [112(38) vs. 132(40), mean(SD) *p* = 0.02].

Patients in the two groups were similar in terms of incidence of opioid related side effects, nausea/vomiting, sedation and pruritus. No adverse events thought to be associated with dexamethasone administration were detected.

## Discussion

The most important finding of this study is that single low dose of dexamethasone administered preoperatively has a significant analgesic effect in the early postoperative period after operative fixation of FNF.

Dexamethasone prophylaxis has been used in dental surgery [13] and thyroidectomy [14, 15], and shown to be a safe and effective method to reduce significantly the postoperative analgesic requirement. Similar doses of dexamethasone administered intravenously preoperatively decrease total analgesic requirements in patient undergoing total laparoscopic hysterectomy [16, 17]. In a recent meta-analysis by Lunn and Kehlet, 17 studies with data from 1081 patients were included and highlighted that high dose of dexamethasone have analgesic effect after hip and knee replacement [18]. De Oliveira et al. included 24 randomised clinical trials with 2751 patients in a meta-analysis. There conclusion were similarly, dexamethasone at doses more than 0.1 mg/kg is an effective adjunct in multimodal strategies to reduce postoperative pain and opioid consumption after surgery [19]. Prior to this study, the potential analgesic benefits of preoperative dexamethasone to patients undergoing fixation of FNF had not been studied. We believe that such benefits as we have demonstrated have particular significance in this population in view of their possible contribution to rehabilitation, a critical determinant of overall outcome in this setting. Although we did not follow patients in this study to identify subsequent benefits of preoperative dexamethasone or persistent post-surgical pain (PPSP), we and others have demonstrated that the quality of early postoperative pain relief is a consistent and important determinant of the incidence of PPSP [20].

In the dexamethasone group one patient’s preoperative saliva cortisol level was greater than the normal range and the postoperative pain scores were higher than the mean at each time point. This might raise the possibility of patients with high preoperative cortisol levels not benefiting from preoperative low dose dexamethasone. This can be only a theoretic explanation so further research is needed in this field.

Our study has certain limitations. The result is positive in terms of primary outcome; however data were studied from only 13 patients in the dexamethasone group because four patients were excluded. This could have contributed to relative under-powering and Type II error in secondary outcomes. We reported no dexamethasone related side effects. The study was not powered to detect clinically meaningful difference, in terms of dexamethasone related side effects, with regard to safety. Our sample size was small but the study was constructed to demonstrate the analgesic effect of dexamethasone, which may didn’t appeared from the pain scores but in the tendency of lower cumulative morphine consumption.

## Conclusions

Our findings indicate that single dose of dexamethasone 0.1 mg kg^−1^ administered immediately preoperatively confers significant analgesic benefit in the early postoperative period after the operative fixation of fractured neck of femur. We have also demonstrated that the patients’ saliva cortisol level were lesser on the first postoperative evening, suggesting an inhibitory effect of the dexamethasone on the hypothalamic-pituitary-adrenal axis.

## Abbreviations

FNF: Fractured neck of femur
NRS: Numeric rating scale
PPSP: Persistent post-surgical pain
SD: Standard deviation
